# Supplementary material for: Phylogeography as a Proxy for Population Connectivity for Spatial Modeling of Foot-and-Mouth Disease Outbreaks in Vietnam
Source: Viruses. 2023 Jan 29;15(2):388. doi: 10.3390/v15020388 (PMC9958845; doi:10.3390/v15020388)
Supplement: Supplementary file 1 [file viruses-15-00388-s001.zip › viruses-2117018-supplementary.pdf]

Table S1. Results from the discrete trait analysis.

From	To	Bayes factor
Pig	Cattle	0.511
Pig	Buffalo	0.753
Cattle	Buffalo	77512.17
Cattle	Pig	77512.17
Buffalo	Pig	3.49
Buffalo	Cattle	2.67

Table S2. Variable selection from the univariate analysis.

From	To	p value	Chi Square	OR
Malaysia	Cambodia	1.45E-07	27.65	inf
Malaysia	Laos	2.20E-16	366.34	inf
Malaysia	china	1	0	1
Malaysia	Thailand	2.20E-16	283.3	inf
Malaysia	Border	2.20E-16	138.71	NA
Malaysia	Slaughterhouse	2.50E-04	13.36	0.43
Cambodia	Laos	3.71E-13	52.79	inf
Cambodia	China	5.96E-14	56.38	inf
Cambodia	Thailand	2.20E-16	68.5	inf
Cambodia	Border	2.20E-16	82.3	NA
Cambodia	Slaughterhouse	9.60E-04	10.9	0
Laos	China	2.20E-16	70.88	3.85
Laos	Thailand	3.13E-15	62.18	3.53
Laos	Border	2.20E-16	177.12	NA
Laos	Slaughterhouse	0.8142	0.05	0.92
China	Thailand	2.20E-16	92.96	0.2
China	Border	2.20E-16	76.36	NA
China	Slaughterhouse	1.25E-12	50.41	7.97
Thailand	Border	2.20E-16	186.9	NA
Thailand	Slaughterhouse	1.61E-09	3.64E+01	0.22
Border	Slaughterhouse	6.56E-08	36.27	NA

Table S3. Results from the univariate analysis.

Variable	Space time DIC	Phylo time DIC
Malaysia	112610.45	110374.64
Cambodia	112429.72	110446.79
Laos	112829.54	110547.01

China	112640.54	110164.08
Thailand	112580.43	110257.4
Border	112846.95	110142.43
Slaughterhouse	112382.32	110074.45
Goat	112758.47	110572.4
Pig	112537.39	110521.59

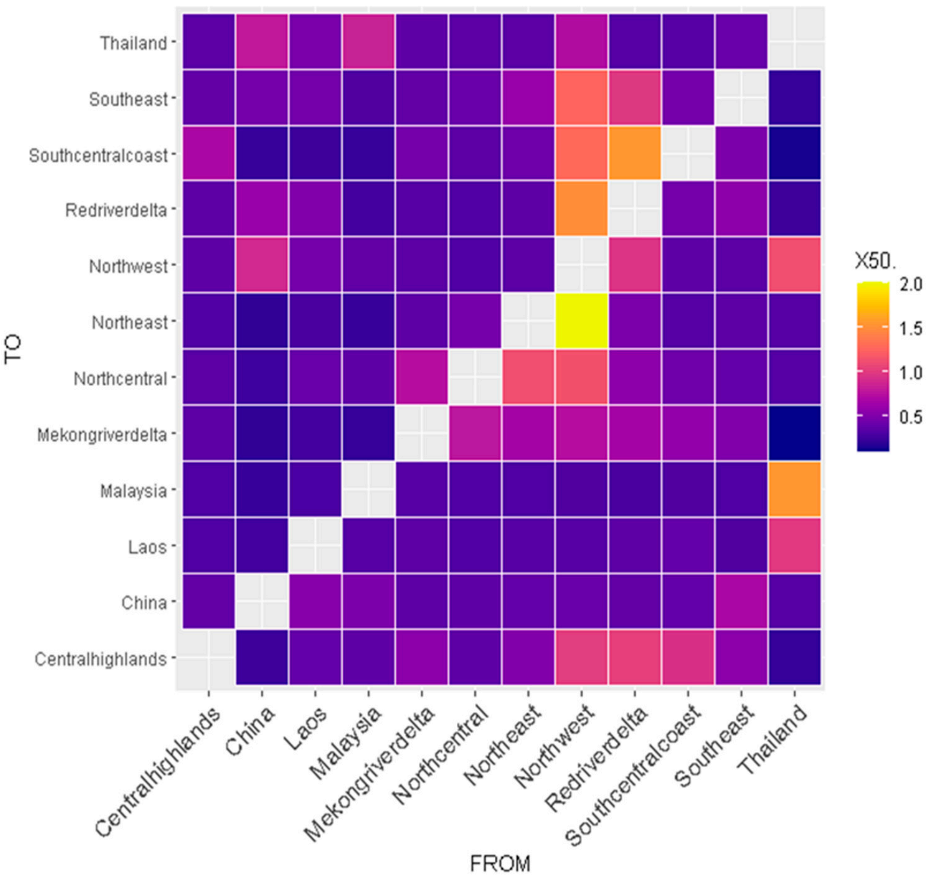

Figure S1. The adjusted rate matrix for Mya 98 lineage showing the virus movement between the different agricultural zones in Vietnam and the adjacent countries. The color gradient of the heat map indicates the adjusted rates, colors closer to yellow shows higher adjusted rates/higher movement compared to purple, which indicate lower adjusted rates/less movement.

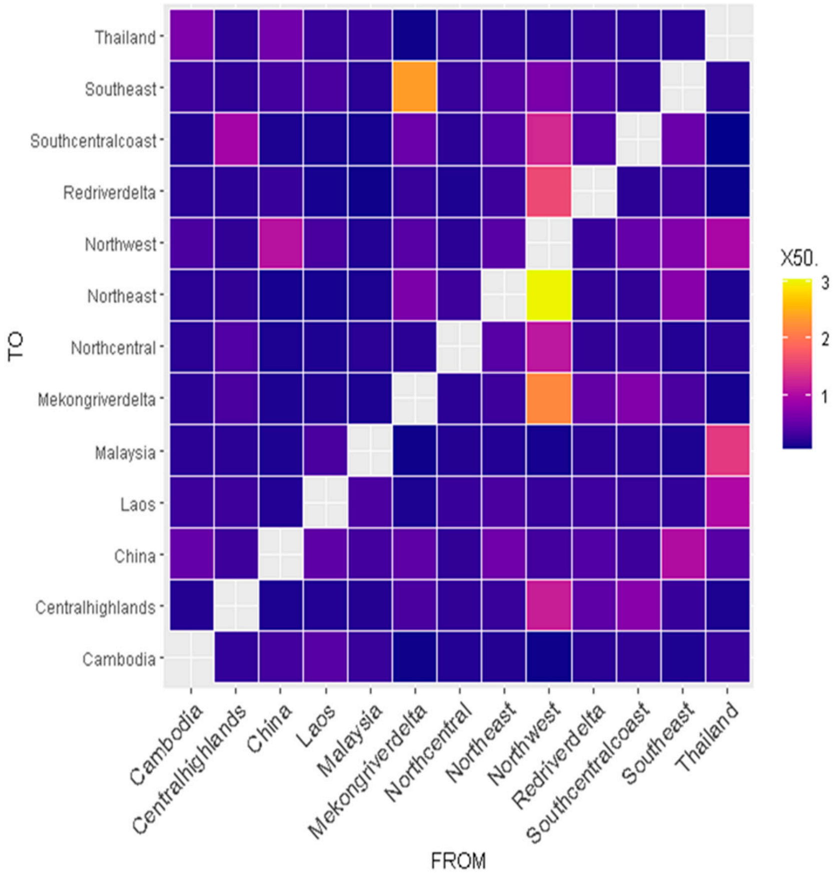

Figure S2. The adjusted rate matrix for total sequences showing the virus movement between the different agricultural zones in Vietnam and the adjacent countries. The color gradient of the heat map indicates the adjusted rates, colors closer to yellow shows higher adjusted rates/higher movement compared to purple, which indicate lower adjusted rates/less movement.

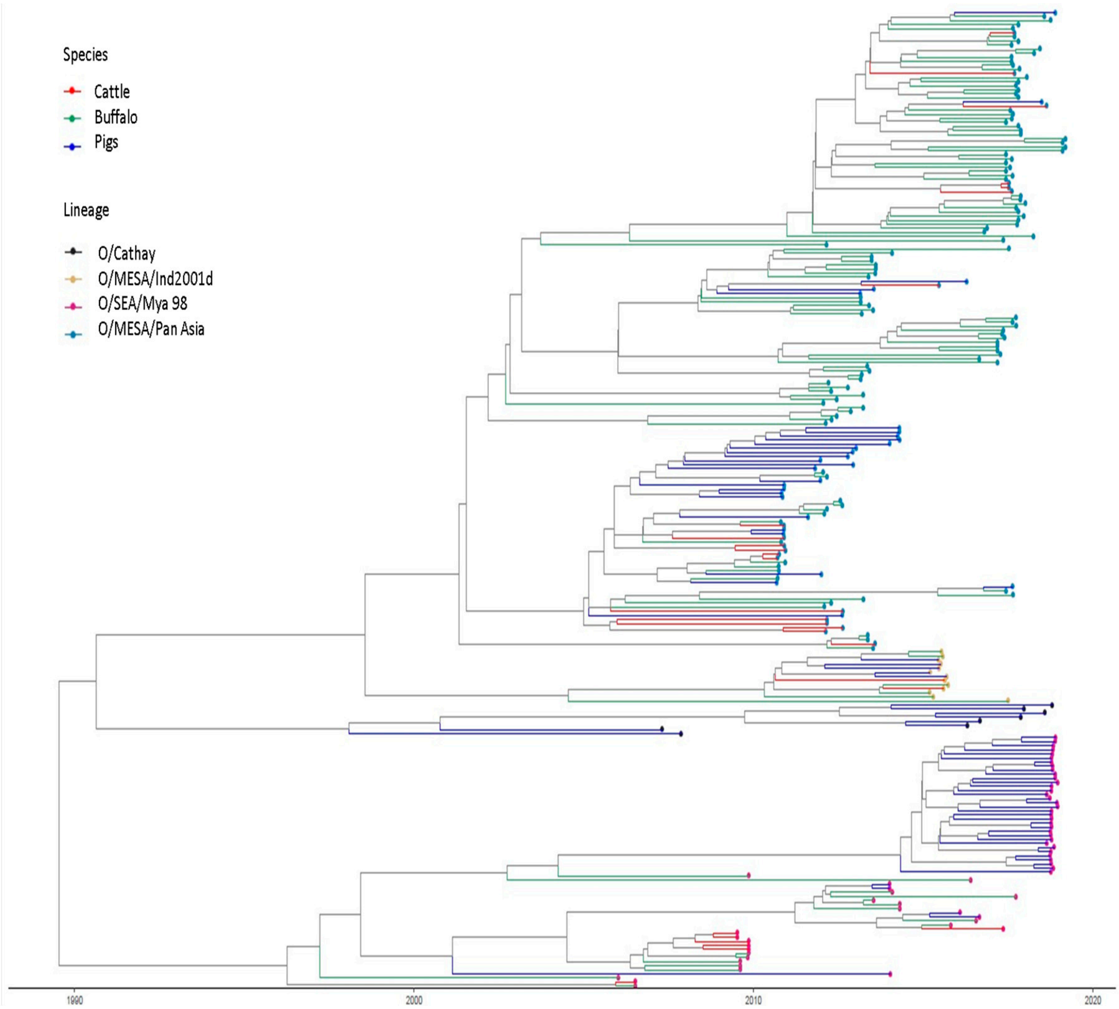

Figure S3. Maximum clade credibility tree for discrete trait analysis BEAST tree from the species analysis. Nodes are colored by the lineage and branches are colored with the species (cattle, buffalo, pig).

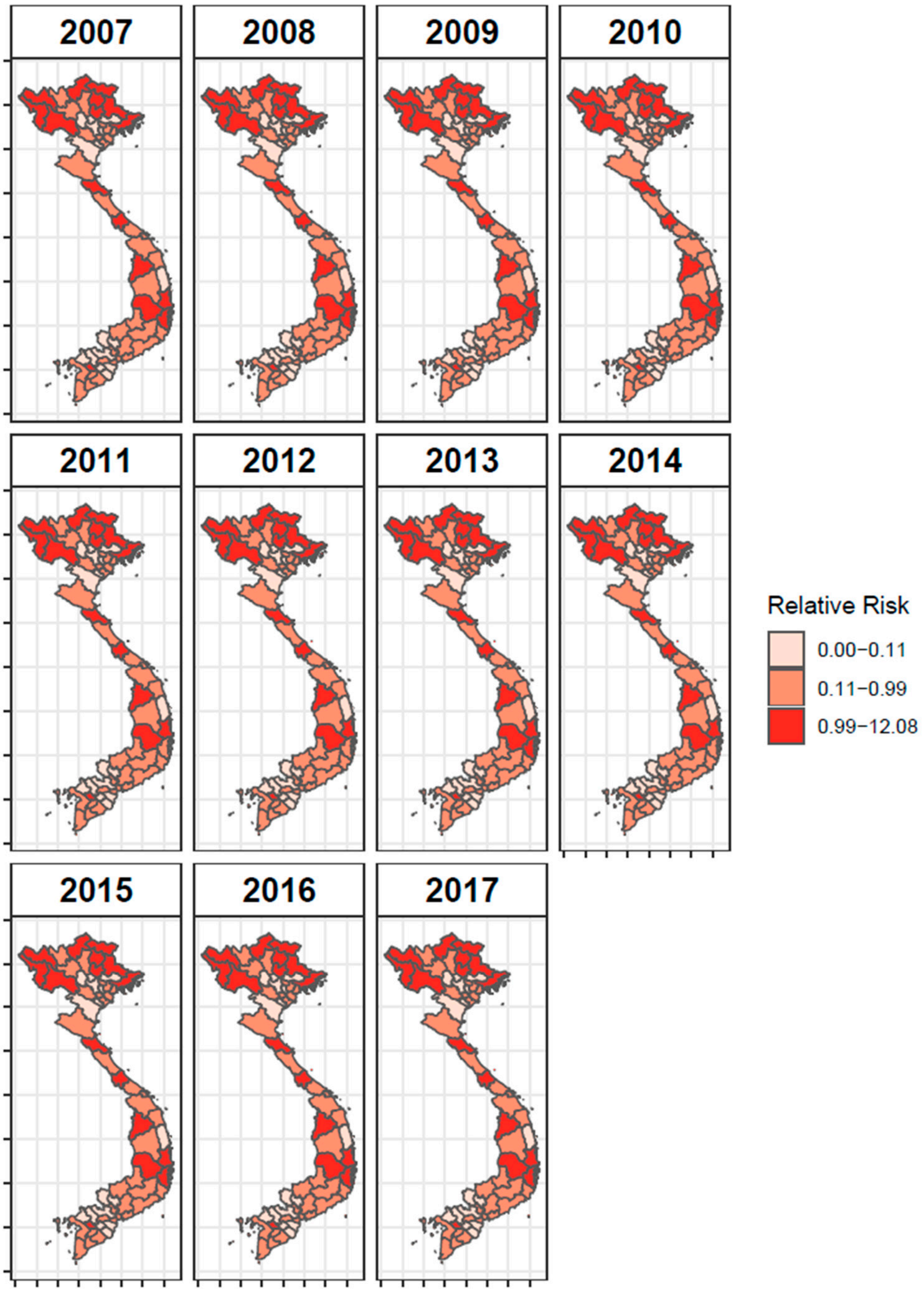

Figure S4. Map of fitted relative risk of outbreaks for each state from the best-fit phylo time model for years 2007-2017 in Vietnam considering the reported outbreak numbers of cattle and buffaloes. Darker areas indicate high risk provinces during the study period.

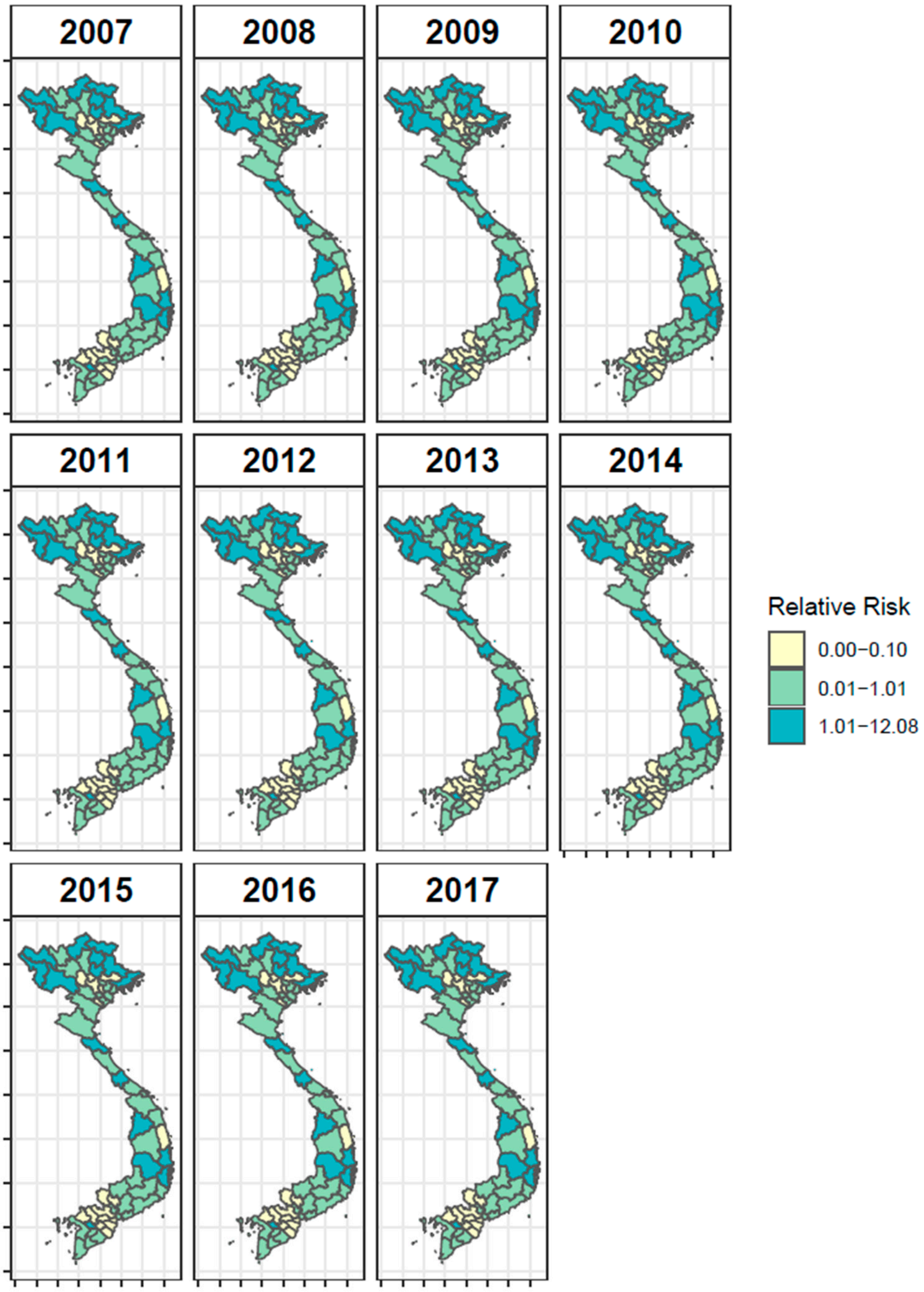

Figure S5. Map of fitted relative risk of outbreaks for each state from the best-fit space-time model for years 2007-2017 in Vietnam considering the reported outbreak numbers of cattle and buffaloes. Darker areas indicate high risk provinces during the study period.

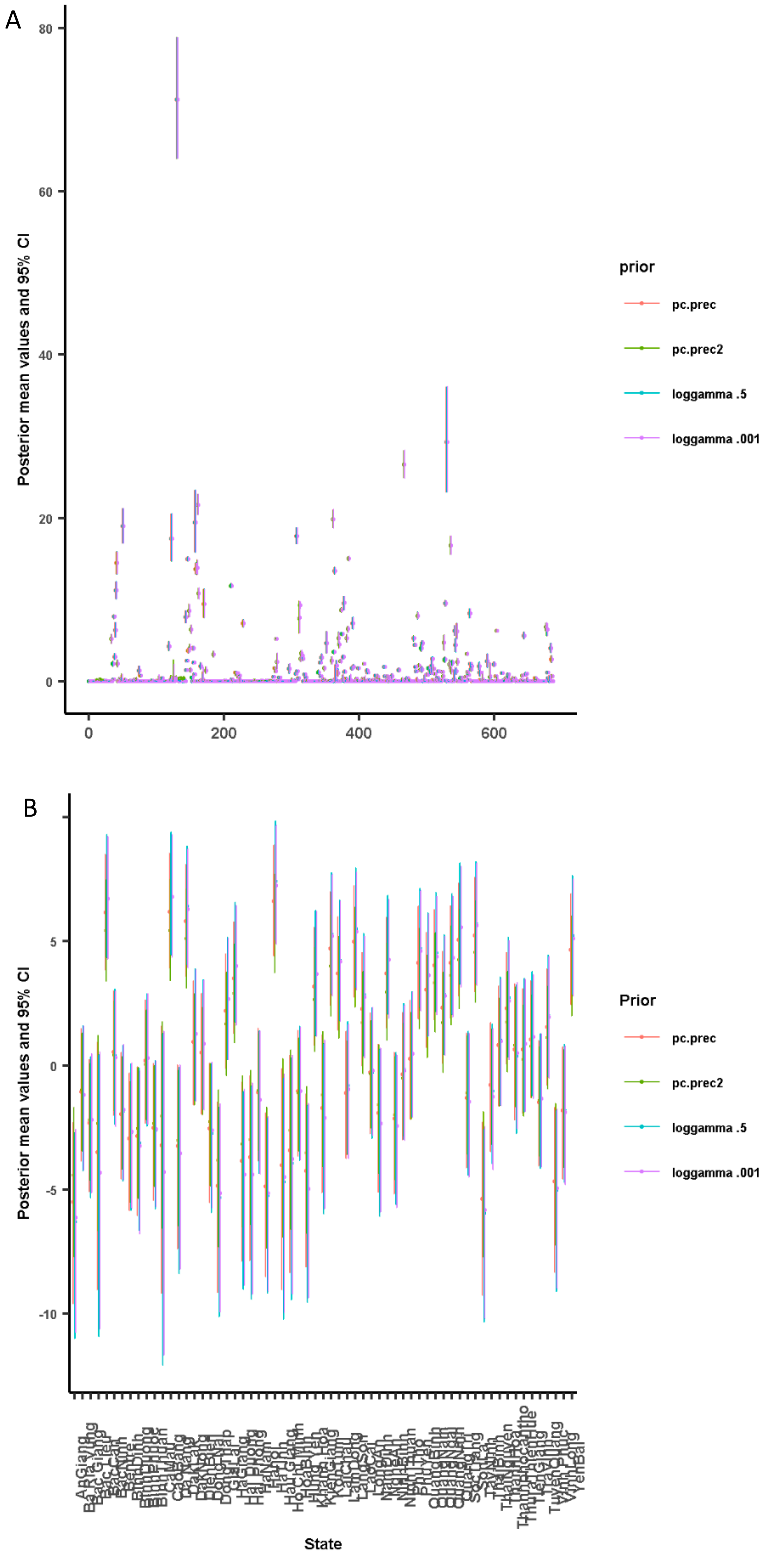
A
Posterior mean values and 95% CI
80
60
40
20
0
0
200
400
600
prior
pc.prec
pc.prec2
loggamma .5
loggamma .001
B
Posterior mean values and 95% CI
5
0
-5
-10
State
Prior
pc.prec
pc.prec2
loggamma .5
loggamma .001

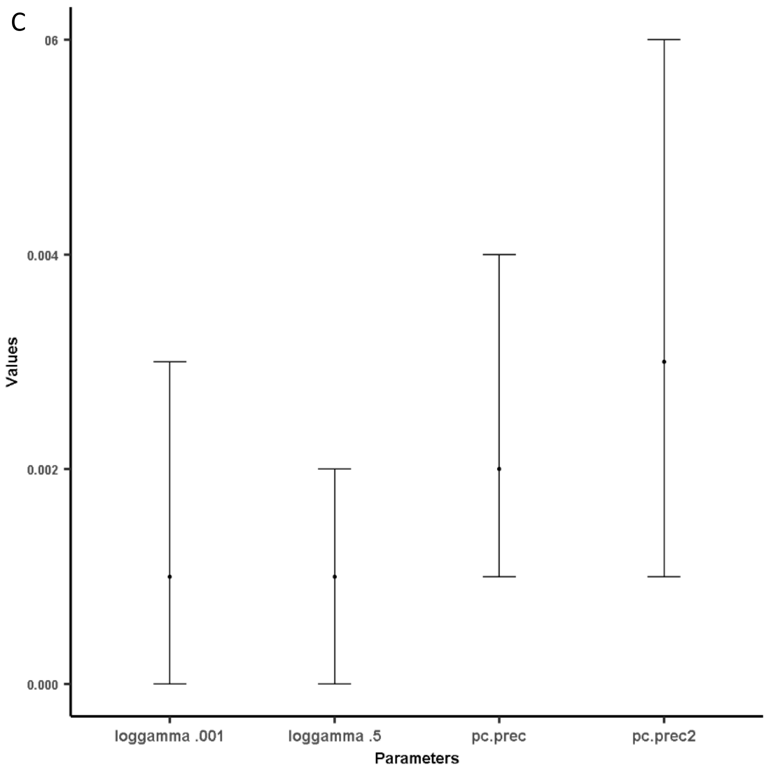

FigureS6. Results from the prior sensitivity analysis showing that different prior combinations produce similar results. (**A**) mean, (**B**) Random effect and (**C**) fixed effects.
